# Associations among Food Systems, Food Environments, Food Choices, Food Security, and Nutrition Transition in Limpopo Province, South Africa: A Cross-Sectional Study

**DOI:** 10.3390/ijerph20166557

**Published:** 2023-08-10

**Authors:** Vhushavhelo Nedzingahe, Betrand Ayuk Tambe, Mthokozisi Kwazi Zuma, Xikombiso Gertrude Mbhenyane

**Affiliations:** 1 Division of Human Nutrition, Faculty of Medicine and Health Sciences, Stellenbosch University, P.O. Box 241, Cape Town 8000, Western Cape, South Africa; 18510663@sun.ac.za (V.N.); ayuk.betrand@yahoo.com (B.A.T.); mkzuma@sun.ac.za (M.K.Z.); 2 Department of Public Health and Hygiene, Faculty of Health Sciences, University of Buea, Buea P.O. Box 63, Cameroon; 3 Impact and Partnerships Division, Agricultural Research Council, P.O. Box 8783, Pretoria 0084, Gauteng, South Africa

**Keywords:** food environments, food security, dietary intake, nutritional status, household, South Africa

## Abstract

A cross-sectional study was applied to investigate the influence of food systems and food environments on food choices and nutrition transition of households in Limpopo province, South Africa. A sample of 429 households was systematically selected using a paper selection draw from three districts. This paper determines the association among food systems, food environments, food choices, and nutritional measurements of the respondents. A validated questionnaire was used. Most of the respondents responsible for food procurement were females (80.4%). There was a significant association (*p* < 0.001) between proximity to food stores and dietary diversity of the households. Staple foods such as bread, maize, rice, samp, and mealie rice were available in almost all surveyed households (95.6%). More than half of the households (59.8%) had home gardens in their yards. Almost half of the households (48.4%) had a low Dietary Diversity Score. The study further revealed that 46.0% of households were food secure. Over a third (36.2%) of the respondents were obese, and 32.5% were diabetic. The mean total blood cholesterol was 3.69 ± 0.74 mmol/L. A high percentage of both females (89.6%) and males (91.5%) had normal hemoglobin levels. Almost half of the respondents had normal systolic blood pressure levels (45.6%), and nearly a quarter had high diastolic levels (21.4%). The 25 coping strategies were applied during food shortage periods. Even though the food environments provided both obesogenic and protective foods, the consumption of unhealthy foods was high.

## 1. Introduction

The ability of the already under-pressure food system to meet the needs of a growing population is further compromised as the effects of human activities are having unprecedented impacts on the earth and its systems through greenhouse gas emissions, which are linked to climate change [1,2]. This has been evident in Tanzanian pastoralists, as climate change has impacted their livestock health, contributing to a reduction in milk production, malnourished livestock, and an increase in cattle deaths, and decreased availability of indigenous fruits and vegetables [3]. Climate change is contributing to increasing food insecurity and preventing the achievement of Sustainable Development Goals (SDGs) [4]. The food system includes the whole extent of activities, people and institutions involved in the production, processing, distribution, marketing, consumption, and disposal of food [5].

The food environment includes a range of food sources and products that surrounds people daily [6]. According to several researchers [7,8,9], the food environment interacts with the food system, as it influences a person’s food procurement and consumption, and these interactions include dimensions, such as the availability, accessibility, affordability, desirability, convenience, marketing, and properties of food sources and products.

The consumption of foods high in sugar leads to obesity and weight gain, which are both risk factors for non-communicable diseases (NCDs). The Global Panel on Agriculture and Food Systems for Nutrition (GLOPAN) [10] reported that approximately three billion people have low-quality diets in 195 countries, and ref. [11] adds that these are steered by an unhealthy food environment. Willett et al. [12] claim that low-quality diets with a lot of red meat and starchy vegetables with minimal fruit and other vegetables, result in micro-nutrient deficiencies and cause a high increase in the incidence of nutrition-related NCDs. Taruvinga et al. [13] reported that dietary diversity in poor populations in the developing world is a significant problem since most foods are starch-rich staple foods, with inadequate animal source foods, fruits, and vegetables. Similarly, Ochieng et al. [14] reported an inadequate consumption of meat, poultry, fish, fruit, and vegetables in most households, which lacks diversity. Moreover, ref. [15] investigated whether dietary quality is associated with socioeconomic status in Saharawi refugee adult population. Their findings indicate that dietary diversity scores are a useful indicator of a household’s socioeconomic status, and nutritional status improves as the dietary diversity score increases [15].

The consumers’ food choices are shaped by the supermarkets and the supply structure of the South African food environment, which is dominated by international companies. It is worth noting that supermarkets currently dominate the food retailing sector in South Africa (SA). This, however, threatens food and nutrition security and the right to food, as supermarkets have reshaped the food system unintendedly and, consequently, influenced nutrition [16]. Evidence shows that over 60% of SA population living in urban areas consumes mostly processed foods, such as bread, sweets, biscuits, alcoholic drinks, carbonated beverages, cheeses, and canned foods [17]. Similarly, ref. [18] revealed that 27.6% of adults aged 19 to 30 years consumed fast foods two to three times a week, with burgers at 69.5%, pizza at 56.6%, fried chicken and soft drinks at 38.4% and 56%, respectively [18]. All this paints a clear picture of the consumption of fast foods, which is alarming.

SA is no exception, as Pillay-van Wyk et al. [19] reported that the country is in a health and nutrition transition. SA faces a unique quadruple disease burden, as described in the first National Burden of Disease study in 2000, and this is despite being an upper middle-income country [20]. To make matters worse, over recent years, the population has been increasingly facing a double burden of malnutrition that includes undernutrition and overweight, obesity, and nutrition-related NCDs [21]. Poor diets and sedentary lifestyles are some of the factors that contribute to the increasing rates of overweight, obesity, and NCDs in the country. This is evident even in Limpopo Province, one of the prime rural provinces in SA, wherein commercial and subsistence farming are highly practiced and contribute to the South African and international food systems. Still, despite this, most households experience some form of food insecurity [22], which is coupled with a high prevalence of stunting, wasting, overweight, and obesity [23]. Due to the nutrition transition, there is public concern about the impact that poor nutrition has on human health [24,25]. This has been recently apparent in African and Asian countries, and it is leading to a higher prevalence of NCDs and a double burden of malnutrition [26,27].

Nutrition transition includes an increase in the consumption of meat and ultra-processed fast and street foods, sugar-sweetened beverages, and animal oils. This study aimed to investigate how food systems and food environments are associated with food choices, food security, and nutrition transition. Food systems, food environments, and food choices are intertwined, and they influence health outcomes, nutrition transition, and food security. Chen and Antonelli [28] identified and categorized key determinants of general food choice, including food-internal factors (sensory and perceptual features), food-external factors (information, social environment, physical environment), personal-state factors (biological features and physiological needs, psychological components, habits, and experiences), cognitive factors (knowledge and skills, attitude, liking and preference, anticipated consequences, and personal identity), as well as sociocultural factors (culture, economic variables, political elements) [2]. Moreover, possible directions of influence among the factors towards final food choice are multiple and complex.

Downs et al. [2] highlighted that the food environment is a critical place in the food system to implement interventions to support sustainable diets and address the global syndemic of obesity, undernutrition, and climate change. Developing countries like SA are also impacted by the syndemic. Food environments contain the total scope of options within which consumers make decisions about which foods to acquire and consume. To integrate linkages between food environments and sustainable diets, food environment interventions should support sustainable diets to enhance human and planetary health. Rosenzweig et al. [29] concur that dietary change in the EAT Lancet report raised awareness of the role that dietary choices can play. Thus, more effective interventions are required to address pressing health concerns and climate change. Noort et al. [30] and others [31,32] predicate that we should change the narrative to empower people to invest in their nutrition for a healthy life, eating food that is supplied by a sustainable food system. Nicoletis et al. [33] and Valentini et al. [34] stated that globally, we need a food system that produces healthy foods for all and prepare for environmental stresses, especially climate-related stresses that lie ahead.

Many researchers agree that we need a food system that produces healthy foods for all, and can withstand environmental stresses, especially climate-related stresses like floods. Minimizing environmental impacts and prioritizing the production of nutritious foods are essential qualities of a sustainable food system. Many African countries have undergone dietary and nutrition transitions in the name of the Western diet and fueled by globalization, rapid urbanization, and development [35,36]. These changes have altered African food environments and, subsequently, dietary behaviors, including food acquisition and consumption. Consumers are influenced by advertising and what they perceive to be modern foods. It is clear that we should reduce meat consumption in favor of legumes, fruits, and vegetables. Determinants of food choice are broad and complex and include policies (government), prices (government and food industry), settings and context (household), as well as interpersonal factors (individual), such as family, cultural, and peer effects [37]. This study attempted to understand the nexus among food systems, food environments, food choices, food security, and nutrition transition.

The study has the following hypotheses:

**Hypotheses** **1** **(H1).** *Food systems and food environments negatively influence the respondents’ food choices, food security, and nutritional status.*

**Hypotheses** **2** **(H2).** *Food systems and food environments positively influence the respondents’ food choices, food security, and nutritional status.*

**Hypotheses** **3** **(H3).** *Food systems and food environments do not influence the respondents’ food choices, food security, and nutritional status.*

## 2. Materials and Methods

### 2.1. Study Design and Setting

The study was a descriptive cross-sectional study with an analytical component utilizing a quantitative approach. The study was conducted in Limpopo province of South Africa, with an estimated total population of 5,852,553 and covers an area of 125,754 km^2^ [38]. Limpopo province has five district municipalities, which are further divided into 21 local municipalities. The population of the province consists of several ethnic groups distinguished by culture, language, and race. The province is dominated by 97.3% of the black population, 2.4% White, 0.2% Colored, and 0.1% Indian/Asian. The study took place in three districts covering three towns, three townships, and three villages.

### 2.2. Target Population, Sample Size Calculation, and Sampling Technique

The target population was households in the different districts of Limpopo province. The sampling approach that was used is multi-phased sampling. Three districts: Capricorn, Mopani, and Vhembe, were purposively selected because of the vast farming activities that enabled a good mix of urban and rural areas. A town, township, and village were selected randomly from each of the three local municipalities by using a random paper draw. Systematic sampling was used to select the households. Every fifth household was selected. In the households, judgment sampling was used to select the respondent who was the adult responsible for food procurement. The estimated sample size was calculated using Slovin’s formula as shown:

Equation:

*n* = N/(1 + Ne^2^)

*n* = sample size

N = population size (number of households)

e = margin of error (0.05)

Therefore, *n* = (1,418,102)/(1 + 1,418,102 × 0.05^2^)

   = (1,418,102)/3546.255

   = 400 households 

A total sample size of 429 households was achieved using a confidence level of 95% and an error margin (0.05).

### 2.3. Data Collection Tool and Procedure

**Figure 1:**
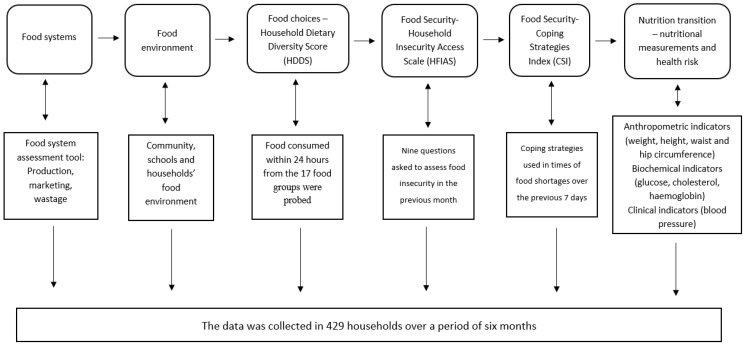
Framework of variables measured, and procedures used.

The developed questionnaire gathered information on the socio-demographic profile and biophysical environmental profile of households, food systems, and food environment, Household Dietary Diversity Score (HDDS), Household Food Insecurity Access Scale (HFIAS), Coping Strategies Index (CSI), nutritional measurements, and health risk assessment, as shown in Figure 1. The food environments and food systems questionnaire were adapted from the nutrition environment measure surveys (NEMS) that have the NEMS-P (used to survey home food environments, the neighborhood, and individuals) [39], NEMS-R (used to survey restaurants) [40], NEMS-S (used to survey food stores) [41] questionnaires. Standardized HDDS [42], adapted CSI [43], and an HFIAS [44] were used.

The current study ensured that the questions in the research instrument cover all the objectives of the study. A pilot test was conducted to test the reliability and effectiveness of the questionnaire, and amendments/changes were inputted. The questionnaire was pre-tested in terms of length (time to complete) and content. After the pre-testing, the research team divided the questionnaire into three sections, which were to be completed during two different data collection visits after an initial visit of recruiting and consenting. Overall, three visits were made to the households on different days. Amendments were made and some questions were removed as they were not relevant based on the views of experts in the field. This ensured the validity of the questionnaire for the study.

Household recruitments were done by fieldworkers, and the study’s aim was explained. An information leaflet was distributed, and those who agreed to participate signed consent forms. The data collection was done by five well-trained research assistants, including the researcher, with the assistance of five to six fieldworkers (FWs) per municipal area. Developed food environment and food system questionnaires, standardized HDDS, CSI, and HFIAS, were administered in the households by the researcher and research assistants. The questionnaire was available in English and three local languages (Tshivenda, Xitsonga, and Northern Sotho) in the province, and the respondent chose the language depending on comfortability. Interviews were conducted using a questionnaire that gathered information on the socio-demographic profile and biophysical environmental profile of households, food systems and food environment, HDDS, CSI, HFIAS, nutritional measurements, and health risks. The 17 food groups HDDS questionnaire was used to determine the food choices in households. The respondents indicated the foods eaten 24 h before the interview. Household food security was determined using HFIAS. CSI was used to assess food availability in households. The respondent interviewed was the adult responsible for food procurement. Figure 1 shows the framework of how the variables were measured.

### 2.4. Ethical Clearance

Ethics approval was granted under reference no: N19/08/112 by Stellenbosch University Health Research Ethics Committee (HREC). Permission was also sought from the relevant districts, municipalities, tribal authorities, and respondents.

### 2.5. Data Analysis

This study analyzed the food system, community food environment, schools’ food environments and household food environments, and their influence on food choices, food security and nutrition transition using IBM Statistical Package for Social Sciences (SPSS) version 27 (IBM Corp., Armonk, NY, USA). Descriptive and inferential statistics were generated for socio-demographic information, the biophysical environment of the households, food systems, food environments, food choices, food security, and nutritional measurements and health risks. Bivariate and multivariate analysis was done to determine the correlations between the variables. For HDDS, the food groups were grouped as poor (<3), low dietary intake (4–7), medium (8–11), adequate intake (12–13), and excellent dietary intake (14–17) [45]. The CSI and HFIAS were used to assess the households’ food security status. In HFIAS, a score of 0 indicated food security, 1–4 indicated mild food insecurity, 5–8 indicated moderate food insecurity, and a 9 indicated severe food insecurity [44]. The CSI was reported in the frequency of occurrence over seven days as a percentage of the total sample [46].

The households’ anthropometric, biochemical, and clinical indicators were used to assess health risk. Waist and hip ratio circumference (WHR) were used to show their nutritional status and identify chronic disease risk. Disease risk is defined when waist circumference (WC) is >88 cm for women and >102 cm for men, to be at risk of metabolic complications [47]. A high WHR was >0.86 cm for women and >1.0 cm for men. Body Mass Index (BMI) is a ratio also known as the Quetelet’s index (height-weight index). BMI classifications were used to assess the weight status of the respondents. BMI was interpreted using WHO [48] in Table 1 below.

A blood sample for a random non-fasting plasma glucose test can be taken at any time. Random blood levels were categorized as normal when less than 11.1 mmol/L and diabetic when >11.1 mmol/L for males and females [48].

Total blood cholesterol levels were categorised as normal when below 5 mmol/L and at high risk of diseases when 7.5 mmol/L or higher for males and females [49].

Hemoglobin normal levels were >12 and >13 g/dL for females and males, respectively. At risk of anemia, levels were <11 g/dL for females and <12 g/dL for males [50].

Blood pressure was categorized as normal blood pressure when the systolic was less than 120 mmHg and diastolic was less than 80 mmHg. At risk of high blood pressure was categorized as systolic being above 120–139 mmHg, while it was high at above 140 mmHg, and diastolic was at risk at 80–89 mmHg or higher at above 90 mm Hg or higher [51].

## 3. Results

### 3.1. Socio-Demographic Information and Biophysical Environment

The findings indicated that about half of the respondents (49.2%) were aged 18–35 years. A majority (80.4%) of the respondents were females (Table 2). More than half of households (61.8%) did not have an elderly person living in the household.

Water and sanitation were assessed, and 53.6% had a water tap in the yard but not in the house, while 43.6% had water taps inside of the house. A total of 51.3% of households used a flushing toilet. Over half of the households (55.3%) used municipal services for the disposal of waste, with 28.9% burning their waste. The total monthly household income of 27.3% ranged from 53.1 to 159.3 US dollars (USD) (1000–3000 South African Rand (ZAR)). Over a quarter of the households indicated that they spent 26.5–53.1 USD (500–1000 ZAR) on food monthly, and only a few (12.6%) spent more than 132.7 USD (2500 ZAR).

### 3.2. Food Systems Assessment

**Figure 2:**
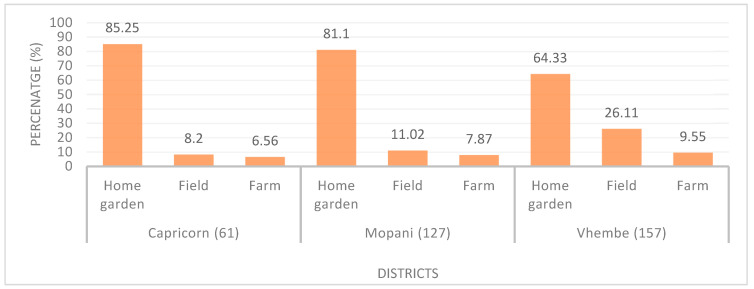
Home garden, field, and farm ownership (*n* = 345).

Overall, 80.6% of households had home gardens, fields, or farms. Figure 2 revealed that most of the households in the Capricorn district had home gardens (85.25%, *n* = 52) in their yards. Only 6.56%, 7.87%, and 9.55% of households had farms for subsistence and/or commercial purposes in Capricorn, Mopani, and Vhembe districts, respectively.

The crops that were mostly planted in home gardens by the households were vegetables (53.4%) and maize (45.5%). Households who reared chickens were 12.4%, and other livestock (1.4%), such as rabbits. More than half of the households (57.6%) consumed the crops they planted, 1.2% sold their crops, and 2.8% both consumed and sold the crops. Over a third (36.6%) of households harvested their maize when still fresh, and 1.9% harvested when it was dry. Over half of the households (55.3%) used municipal services to dispose of waste, with 28.9% burning their waste.

### 3.3. Food Environment Assessment

A high presence of spaza shops was reported by the respondents (88.3%), with open markets/street vendors and convenience/corner stores being reported by 79.3% and 75.5%, respectively. Study respondents (84.1%) also reported a high presence of liquor stores in the communities. A total of 49% and 51.3% of respondents indicated that there were three to seven food and liquor stores in the communities, respectively. The study findings revealed that 93.2% and 88.6% of staple foods were sold in convenience/corner/spaza shops and grocery/supermarkets, respectively. Street vendors mostly sold fruits (79.3%) and vegetables (80.4%). Convenience/corner/spaza shops were reported as being very expensive by nearly half of the respondents (49.2%) as compared to street vendors and grocery stores. Over half of the respondents (50.9%) did not go to restaurants, and almost a third (27.5%) of those that did, indicated restaurants as very expensive.

**Figure 3:**
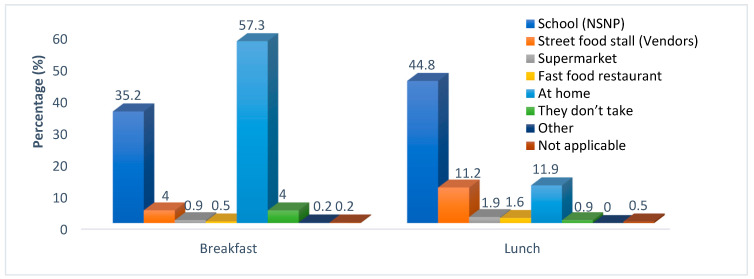
Places of breakfast and lunch of school children.

Fruits (42.0%) and vegetables (47.3%) were more affordable as compared to low-fat products (3.7%). Almost a quarter (24.9%) of the respondents indicated that they did not see any healthy food adverts in stores. Many (77.5%) have seen food discounts in stores, and the main discounted foods were basic foods, such as chicken braai packs, sugar, maize meal, and milk, among others. Almost all respondents (94.4%) indicated that the kinds of foods that are packed near the cashier/tills were chocolates, sweets, and cold drinks. Many of the respondents (79%) indicated that the children’s schools offer facilities to buy food. It was revealed that 57.3% of children had breakfast at home, with only 4% and 0.9% buying food for breakfast from street vendors and supermarkets, respectively, as shown in Figure 3. Almost half (44.8%) of children received their lunch from the National School Nutrition Program (NSNP) at school, while a few (11.9%) got it from home. The study findings showed that 34.8% of children took lunch boxes to school every day. A few respondents (1.3%) indicated that their children took lunch boxes because of their special diet.

**Figure 4:**
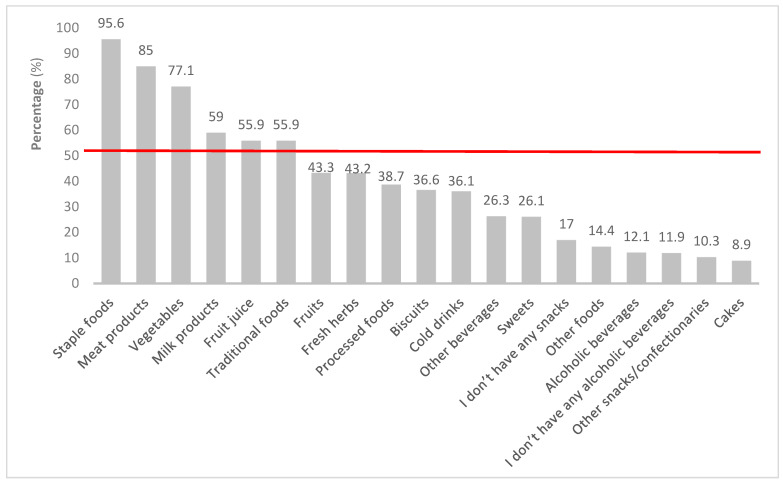
Availability of food in the households on day two of visit.

Figure 4 shows that staple foods were available in almost all surveyed households (95.6%). In more than 50% of households, food that was often available included staple foods, meat products, and vegetables.

The majority (97.9%) of households bought their food from grocery stores or supermarkets and only 5.8% bought their food from spaza shops. Most households (89.0%) bought their food monthly. A total of 83.0% of the respondents mostly considered price when they bought their food. A total of 57.8% of the households always had easy access to vegetables and 40.4% to fruits. It was noted that 72.5%, 70.6%, and 76% of households sometimes consumed fruits, vegetables, and SSBs during the day and at mealtimes, respectively.

Almost half (47.1%) of the respondents were always encouraged to cook or make healthy food choices in the households by other household members. Almost half of the respondents (47.1%) self-reported their nutritional knowledge as average. When asked to give examples of healthy foods, a majority of respondents (88.3%) mentioned “fruits and vegetables” and almost all the respondents (93.5%) mentioned unhealthy foods as “foods with a lot of fat, salt and sugar”. Furthermore, the findings revealed that 74.8% of the respondents listened to the radio and watched television to obtain nutritional knowledge.

### 3.4. Food Choices

Most households in the study cooked daily (79.5%), with 13.5% who cooked only two to four times a week. When asked about meals that were eaten but not prepared at home, 63.6% did not have any such meals, 24.9% had one meal, 11.0% had two to five meals, and those who had >5 meals were only a few (0.5%).

**Figure 5:**
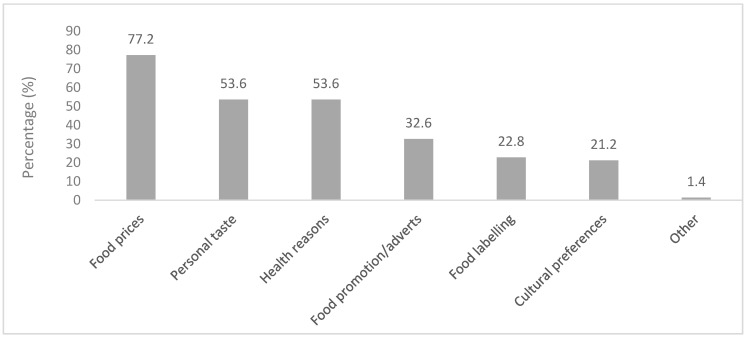
Factors that influence food choice.

Various factors in Figure 5 below were reported to influence the households’ food choices, of which 77.2% of the respondents mentioned food prices as the most influential factor, whereas the least two factors stated were food labeling (22.8%) and cultural preferences (21.2%). Over half of the respondents (50.9%) indicated that they did not go to restaurants, and 49.1% ate out. Most (62.7%) respondents preferred fast-food restaurants, such as KFC restaurants. Restaurant outings were positively and significantly (r = 0.275, *p* = 0.000) associated with the DDS of households.

Cereals (92.8%) and white tubers and roots (31.1%) were the most consumed starches. There was a lower consumption of vitamin A-rich vegetables (37.6%) compared to dark green leafy vegetables (45.3%). Almost half of the households consumed other fruits (45.3%), and just over a third (30.8%) consumed vitamin A-rich fruits. Organ meat (iron-rich) (30.4%) and red palm oil (21.3%) were the least consumed food groups.

**Figure 6:**
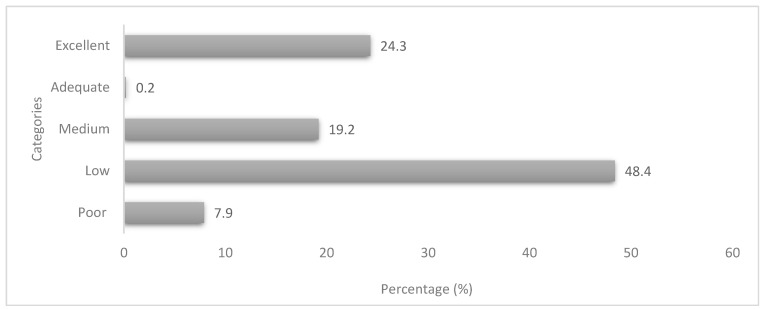
Household dietary diversity classifications.

Figure 6 shows that almost half of the households (48.4%) had a low DDS score (score of 4 to 7), and 19.2% had a medium score (score of 8 to 11). Households that had an adequate score (score of 12 to 13) were 0.2%, and those with an excellent score (14–17) were 24.3%. A significant association was found between marital status and dietary diversity (r = 0.113, *p* = 0.019). Furthermore, educational level (r = 0.465, *p* = 0.035) had a significant association with dietary diversity. The results showed that proximity to food stores (r = 0.225, *p* = 0.001) was positively and significantly associated with dietary diversity.

### 3.5. Household Food Security Status

The three most used coping strategies by the households were: Rely on less preferred and less expensive foods (31.3%), reduce portion sizes (23.1%), and limit portion size at mealtimes (22.4%), as shown in Table 3. Almost half (45.2%) of the respondents were worried that their households would not have enough food to sustain them in the past month. Some households (15.1%) did not have any kind of food due to the lack of resources or money to purchase the food. The least used three coping strategies were: Feed male members of HH at the expense of female members, feed adult members of HH at the expense of children, and feed working members of HH at the expense of non-working members at 2.1% each.

The current study revealed that 45.2% of households faced anxiety and uncertainty over food, having had worried about the availability of food in the household, and 46.0% had insufficient food intake in the preceding month. This includes eating smaller meals than usual, as shown in Table 4 below. It was noted that 15.6% of households did not have any food due to a lack of resources/money to purchase the food. The average HFIAS score was 4.4 out of a maximum of 27, which means that households rarely faced one of the conditions. HFIAS categories showed that almost half of the households were food secure, with only 4.0% of households experiencing severe food insecurity.

Table 5 below displays the multivariate logistic regression analyses compared with Household Food Insecurity after adjusting for all potential confounders.

### 3.6. Nutritional Status and Health Risk

#### 3.6.1. Anthropometric Indicators of the Respondents

**Figure 7:**
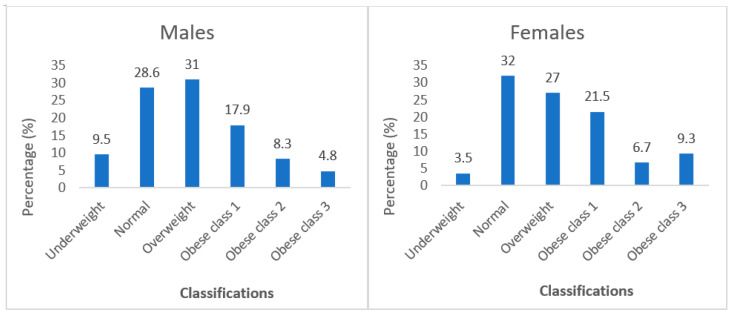
BMI classifications of the respondents by gender.

Overall, thirty (30.3%) of the respondents had a normal BMI (18.5–24.99 kg/m^2^), while 36.2% were obese, which is classified as a BMI of >30 kg/m^2^. BMI status was also determined according to gender, as Figure 7 shows. The BMI results indicate that 31.0% of males were overweight as compared to 27% of females, and 31.0% of males were obese as compared to a higher 37.5% of females. Almost all the males (94.0%) had a normal WHR as compared to the 54.5% of females. Only 16.7% of males were at risk of experiencing metabolic complications as compared to over half of females 53.4%.

#### 3.6.2. Biochemical Indicators of the Respondents

The study found a rate of 32.5% of respondents whose random blood glucose level was greater than 11 mmol/L, as compared to 67.5% who had normal (<11.1 mmol/L) blood glucose. The mean total blood cholesterol was 3.69 ± 0.74 mmol/L, as shown in Table 6. A high percentage of both females (89.6%) and males (91.5%) had normal hemoglobin levels. Only 8.5% of males were at risk of anemia as compared to 10.4% of females.

#### 3.6.3. Clinical Indicators of Respondents

**Figure 8:**
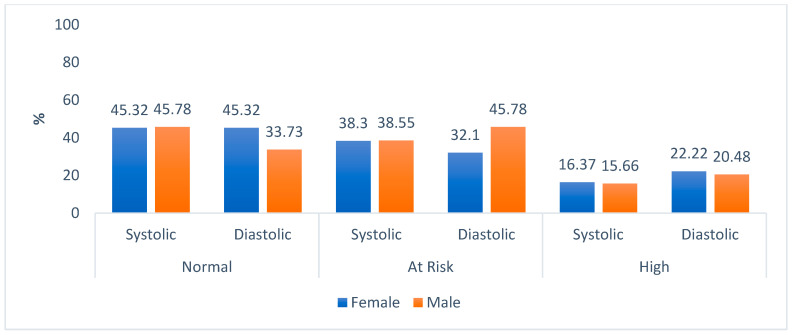
Blood pressure levels of household respondents by gender.

The household respondents who self-reported living with high blood pressure were 17.1%. Figure 8 shows the blood pressure readings in systolic and diastolic levels. It is evident that about the same percentages of females and males had normal systolic levels, 45.32% and 45.78%, respectively. For diastolic levels, more females than males had normal levels (less than 80 mmHg). For high-risk systolic levels, which were >140 mmHg, percentages were similar for both genders, similarly with the diastolic.

## 4. Discussion

The majority of respondents responsible for food procurement were females (80.4%), and only a quarter were males. Females are regarded as nurturers in many households and are primarily responsible for food preparation and shopping, so it makes sense that they are dominant. Approximately two-thirds of households had three to five household members, and almost all the households had children. Similarities were reported by Stats SA [52] as a third of households in Limpopo Province had two to three household members, followed by over a quarter that had four to five household members in 2018. Thus, this shows that most households were of average size. A study by Mkhawani et al. [53] revealed that 37% of the caregivers did not have any tertiary education compared to the 66% in this study. However, nationally, a higher rate was reported by Stats SA [54], as 91.9% of individuals did not attend any tertiary education in 2018. This is alarming and can further exacerbate the inequality in the country. Over a third of the respondents earned less than 159.3 USD (3000 ZAR), similar to what others found as 53.0% of rural formal dwellers reported earning between 42.5–170 USD (801–3200 ZAR) per month [54,55], and this is due to minimal employment opportunities. This income distribution reflects the current situation in SA, where most households rely on government grants to secure food. The findings in this study have been asserted in other studies [13,56,57]. Over half of the households spent less than 80 USD (1500 ZAR) on basic foods monthly, and this is what Ward et al. [58] also found in their study as households resorted to cutting back on food spending for other essentials such as utilities and housing. The Pietermaritzburg Economic Justice & Dignity Group (PMBEJD) gave some insight as to why households spend less on food. The household food basket in SA is close to 265.5 USD (5000 ZAR) [59], which is way higher than the national minimum wage, which is around 185.4 USD (3500 ZAR) per month [60].

Many households in the current study were practicing farming, and these findings align with those of a study by Shisana et al. [23]. The practice of planting crops gave households more options for sourcing fresh produce while enhancing household food security and nutrition. The findings showed that just below a third of the households had livestock such as chickens, cows, pigs, and goats. Vegetable and livestock farming has been associated with increased food security and dietary diversity in other studies [61,62]. However, this study did not find any significant association between crop and livestock farming and dietary diversity in surveyed households. Vegetables, such as spinach, cabbage, mustard, tomatoes, and onions, were the most grown in over half of the households. This was followed by maize, which was grown by 45.5% of households. Findings in a survey conducted by Mullins et al. [63] showed that half of the households grew at least one type of fruit or vegetable in their home garden. Many households reported that they started engaging in home gardening due to the COVID-19 pandemic. Mullins et al. [63] reported that there are links between times of economic hardship and increases in home food gardening. Similar findings were reported by Ogundiran et al. [64] in a study about the role of home gardens in household food security in the Eastern Cape Province. Households that consumed their crops were 57.6%. Only 13.8% of households reared livestock. Many households’ rear livestock traditionally for wealth [65].

The food environment is the interface between food systems and consumers and includes the physical, economic, and socio-cultural factors that influence food choices. “Today’s food environments exploit people’s biological, psychological, social, and economic vulnerabilities, making it easier for them to eat unhealthy foods” [66]. As Lang [67] reported, this is mainly because “food systems are dominated by powerful interest, some of which can be deeply opposed to change, and too often, in battles for policy leverage, the public interest may get lost”. Food environments that expose people to unhealthy food choices prove our food systems are failing to provide an enabling food environment. These unhealthy diets are driving the overweight and obesity crisis globally, which, in turn, lead to chronic diseases. The study showed that just below half of the learners took lunch boxes to school. Similarly, another South African study [68] found only a quarter of learners took a lunch box to school. In contrast, a high number of learners took a lunch box to school in a study conducted in Cape Town [69]. This concurs with South African schools’ policy encouraging learners to carry lunch boxes to limit unhealthy foods and include fruits [68,69,70]. Most food purchases at schools in the present study were mainly unhealthy options, such as fat cakes, sweets, and crisps from street vendors. Similarly, two other South African studies reported learners purchasing sweets, chocolates, and chips from the school tuckshops [71,72]. These food items are generally high in fat, sugar, and salt and are energy-dense, exposing the learners to an unhealthy school food environment.

In terms of the availability of foods in the households, staple foods such as pap (stiff porridge made from maize), bread, and rice were reported in almost all of the households. Kroll et al. [73] agree that most households had maize and bread present. Furthermore, high-energy staple foods such as sugar, sweets, as well as soft drinks, were prevalent. The findings of this study showed that 77.1% of vegetables and 43.3% of fruits were available in the households. In comparison to the current study, Chai et al. [74] found that 85.4% of respondents had fruits available in their homes always or most of the time. More than half of the households had salty snacks, and in terms of the availability of confectionaries in the households, biscuits were the most common, followed by sweets and cakes. It was evident that most households had sugary beverages in their households as compared to a few who had alcoholic beverages. The pattern of consumption of alcohol was different as compared to the Shisana et al. [23] study, which reported almost half of the respondents consumed alcohol. South African consumers, particularly from low-income households, are impacted the most by rising food costs as SA has one of the highest inflation rates for food in comparison to other countries [75]. This is perhaps why a high percentage of households in the current study reported food prices as an influential factor in their food purchasing. Castro et al. [76] concur that food prices affect consumers’ purchase intentions and food choices. Interestingly, the current study did not find any association between food prices and the dietary diversity of households.

A diversified dietary intake improves nutrient adequacy, thus ensuring meeting nutrient requirements and lowering nutritional deficiencies. The food group that was consumed by almost all households was cereals, which included the starchy staples group. The foods mostly consumed were maize-based foods such as soft porridge and stiff porridge in the preceding 24 h. These findings are consistent with those reported [13,77] in rural and urban towns of SA. The current study found that over a third of households consume fruits, including vitamin A-rich fruits. The low consumption of fruits was observed in a study in Cameroon that found that only a few households were consuming fruits [78]. Vegetables were consumed by a majority of the households. This includes vitamin A-rich vegetables and dark green leafy vegetables. However, Chakona and Shackleton [79] found dissimilar findings where a third of respondents consumed such vegetables. The low consumption of fruits and vegetables can lead to inadequate micronutrient intake, which increases vulnerability to food insecurity. Thus, Tambe et al. [80] reported that a higher DDS is associated with improved health.

Dietary diversification is strongly associated with household socioeconomic status [15], that is, the higher the household income, the higher the dietary diversity. It was noted that households with tertiary education had a high monthly household income leading to a high dietary diversity. Furthermore, nearly half of the households had a low dietary intake, whereas just below a quarter was in the excellent group. These results are similar to what Cheteni et al. [57] found, where 60% of households fell into the lower dietary diversity group, and one-fifth was in the high dietary diversity group. In addition, most households consumed three food groups, which included milk, cereals, and pulses. However, the latter contrasts with the current study as most households consumed four to six food groups which included cereals, vegetables, meat, spices, condiments, and beverages and sweets in the form of sugar. Nonetheless, the same conclusion of low dietary diversity holds in this study as well as that of Taruvinga et al. [13], conducted in the rural communities of the Eastern Cape province in SA.

Borrowing from a neighbor is a known African practice dating to centuries ago as people living in rural areas typically live as a closely-knit unit and assist each other with food and other necessities. However, a few households borrowed food from neighbors or purchased food on credit when dealing with food shortages. When probed further, respondents indicated that they would rather stay without food than ask for food from neighbors, which might indicate that African practices are fading out. These findings are contrary to Mbhenyane et al. [55], who found that most households borrowed food from their neighbors, family, or friends and bought food on credit from the local shop to cope with food deprivation. A third of households relied on less preferred and less expensive foods, and almost a quarter reduced their portion sizes. Similarly, findings by Nabuuma et al. [80] showed that 48.6% of the households consumed less preferred foods, and 48.9% limited the variety of foods eaten. The findings showed 54% of households as food insecure, which also holds in a study by [81]. However, other studies [82,83] have shown a much higher prevalence of food insecurity as compared to this study. In contrast, a study conducted by [78] found 50% of households to be food insecure. High unemployment, inadequate food consumption, and poverty are some of the factors that contribute to food insecurity significantly.

Overweight and obesity are a challenge worldwide and are major health risk factors for diseases like diabetes, high blood pressure, CVDs, and some cancers [84]. Over a quarter of respondents were overweight and one-third obese, and it was evident that most foods consumed were processed foods, high-energy foods that contain lots of fats and oils, sugar, and salt, and these contribute to obesity. Cois and Day [85] indicated that the prevalence of obesity in the South African population is increasing, especially among women. This supports Stats SA [54] findings as 31% and 68% of men and women were overweight or obese, respectively. Moreover, people who are overweight or obese tend to have high waist circumference and waist-to-hip ratio [56], especially women. This is evident as over half of female respondents had a high waist circumference, and almost half had a high WHR. Similarly, Shisana et al. [23] found that 47.1% of females had a WHR that was almost seven times greater as compared to the 6.8% of males.

A report by the WHO [86] estimates that 422 million people had diabetes in 2014 worldwide. This is a prevalence of 8.5% among the adult population. A study by Shisana et al. [23] reported a lower prevalence of only 4.6% who were diabetic in Limpopo province. However, another study in the Eastern Cape by Sharma et al. [82] reported over half of the respondents as diabetic. The latter is similar to this study as over a third of respondents were diabetic or at risk of being diabetic (>11 mmol/L), with a similar percentage self-reporting to be diabetic. The prevalence of diabetes has been steadily increasing for the past three decades, and it is evident in LMICs and SA is no exception.

High cholesterol has been linked to increasing the risk of having stroke and heart disease [49]. Respondents who self-reported that they had stroke and heart disease were very few. This holds in the current study with respondents who had high cholesterol. Similarly, a report by Virani et al. [87] revealed that nearly 12% of adults aged 20 and older in the USA had total cholesterol higher than 240 mg/dL in 2015–2018. Stats SA [55] reported a prevalence of anemia among men aged 15 and older as lower than for women (17% compared with 31%). Interestingly, the current study found lower rates of anemia for both males and females, regardless of the lack of dietary diversity.

The prevalence of prehypertension and hypertension keeps rising in SA regardless of the available interventions. According to the National Institute for Health and Care Excellence (NICE) [88], each 2 mmHg rise in systolic blood pressure is associated with a 7% increase in the risk of death from ischemic heart disease and a 10% increased risk of death from stroke. A study by Shisana et al. [23] revealed that respondents with high systolic blood pressure were 38.2%, and 20.0% had high diastolic blood pressure, whereas the current study found higher rates of more than half of both males and females at risk, and those who had elevated systolic blood pressure (>140 mmHg) and high diastolic blood pressure (>90 mmHg). Interestingly, Stats SA [54] reported that Limpopo province has the lowest rates of high blood pressure as compared to other provinces.

The major limitations were the usage of the dietary diversity questionnaire only to determine the food choices of households. Another instrument, such as Food Frequency Questionnaire (FFQ), could have been used to supplement data from the dietary diversity questionnaire. The dietary diversity questionnaire, CSI questionnaire, and HFIAS all probed the respondents to recall foods eaten and behaviors that happened previously. The recall of information can be unreliable and imperfect as it depends entirely on memory. There was a higher distribution of households from rural settings as compared to urban settings due to difficulties in securing urban households, and this might have contributed to the higher rate of food insecurity and low DDS. The results of this study cannot be generalized to the South African population as it was conducted in one out of the nine provinces.

## 5. Conclusions

The study reveals significant findings on the food systems and food environments. The current finding proves that the current food systems and food environments have a negative influence on the populations’ food choices and nutritional status by the following: 1. The presence of food stores in the areas provided access to obesogenic despite the socioeconomic status of the household; 2. The availability of refined, energy-dense food in school environments, such as chips, kota, sweets, and chocolates, instead of fruit and other healthy options was high and is worrisome as learners frequently consume these unhealthy foods. Lastly, 3. DDS was low in most households, with more than half of the households being food insecure. Therefore, the findings prove the study hypothesis that food systems and food environment influenced the food choices and nutritional status of the study population in a negative manner. In light of these findings, the complexity characterizing the South African food system and the resultant negative food insecurity shows that significant changes should be made. The associations between food environments, the role of the food industry, the food choices, and behavior of consumers remain under-researched, so more research should be done focusing on these.

## Figures and Tables

**Table 1:** BMI classifications.

Interpretation	Classifications (kg/m^2^)
Underweight	<18.5
Normal	18.5–24.99
Overweight	25–29.99
Obese class 1	30–34.99
Obese class 2	35–39.99
Obese class 3	>40

Source: WHO [48].

**Table 2:** Socio-demographic information (*n* = 429).

Categories	Frequency (*n*)	Percentage (%)	*p* Values
*Age (years)* *			
18–35	210	49.2	
36–55	156	36.5	<0.001
>55	61	14.3	
*Marital Status*			
Single	248	57.8	
Married	119	27.7	<0.001
Divorced	13	3.0	
Widowed	25	5.8	
Cohabitation	22	5.1	
Other	2	0.5	
*Gender*			
Female	345	80.4	<0.001
Male	84	19.6	
*Education*			
Never attended school	21	4.9	
Primary education (grade 1–7)	26	6.1	
Secondary education (grade 8–12)	232	54.1	<0.001
Tertiary education (degree, diploma, etc.)	146	34.0	
Other	4	0.9	

* *n* = 427 household respondents.

**Table 3:** Coping strategies used by households in times of food shortage (*n* = 429).

Usage		No of Days						
Behaviours	% (*n*)	1	2	3	4	5	6	7
Rely on less preferred and less expensive foods	31.3 (134)	4.7 (20)	8.4 (36)	5.1 (22)	2.8 (12)	2.1 (9)	0.7 (3)	7.5 (32)
Reduce portion sizes	23.1 (99)	4.7 (20)	6.3 (27)	4.0 (17)	3.5 (15)	0.2 (1)	0.0 (0)	4.4 (19)
Limit portion size at mealtimes	22.4 (96)	4.0 (17)	6.8 (29)	3.7 (16)	2.8 (12)	0.9 (4)	0.0 (0)	4.2 (18)
Borrow food, or rely on help from a friend or relative	18.0 (80)	8.4 (36)	4.4 (19)	2.1 (9)	1.4 (6)	0.5 (2)	0.0 (0)	1.2 (5)
Reduce the number of meals eaten in a day	17.7 (76)	4.7 (20)	6.3 (27)	2.1 (9)	0.9 (4)	0.9 (4)	0.2 (1)	2.6 (11)
Not having enough food or money to buy food	15.1 (65)	3.7 (16)	3.5 (15)	3.3 (14)	1.4 (6)	0.2 (1)	0.0 (0)	3.0 (13)
Purchase food on credit	13.7 (59)	6.8 (29)	3.0 (13)	1.6 (7)	1.2 (5)	0.2 (1)	0.2 (1)	0.7 (3)
Skip meals	13.6 (59)	3.7 (16)	5.1 (22)	2.1 (9)	0.9 (4)	0.0 (0)	0.2 (1)	1.6 (7)
Borrow money to buy food from neighbours	12.6 (54)	7.5 (32)	2.6 (11)	0.9 (4)	0.2 (1)	0.9 (4)	0.0 (0)	0.5 (2)
Take other measures	13.3 (51)	4.0 (17)	2.7 (11)	3.7 (10)	0.6 (3)	0.2 (1)	0.0 (0)	2.1 (9)
Drink tea only	11.3 (48)	2.6 (11)	4.7 (20)	1.9 (8)	0.2 (1)	1.9 (8)	0.0 (0)	0.0 (0)
Restrict consumption by adults in order for small children to eat	7.4 (32)	2.8 (12)	2.3 (10)	1.2 (5)	0.2 (1)	0.0 (0)	0.0 (0)	0.9 (4)
Borrow food from neighbours	6.9 (29)	1.9 (8)	2.6 (11)	0.7 (3)	0.7 (3)	0.5 (2)	0.0 (0)	0.5 (2)
Sleep without food	5.5 (24)	3.0 (13)	0.9 (4)	1.2 (5)	0.0 (0)	0.2 (1)	0.0 (0)	0.2 (1)
Send household members to beg	5.1 (22)	1.4 (6)	1.6 (7)	1.2 (5)	0.2 (1)	0.5 (2)	0.2 (1)	0.0 (0)
Send children to neighbours or relatives	5.1 (22)	2.1 (9)	1.9 (8)	0.5 (2)	0.2 (1)	0.2 (1)	0.0 (0)	0.2 (1)
Send household members to eat elsewhere	4.4 (19)	1.2 (5)	1.9 (8)	0.2 (1)	0.2 (1)	0.0 (0)	0.0 (0)	0.9 (4)
Feed female members of HH at the expense of male members	2.5 (11)	1.4 (6)	0.5 (2)	0.2 (1)	0.0 (0)	0.2 (1)	0.0 (0)	0.2 (1)
Exchange sorghum/green mealies for white mealie meals from local shops or mobile vendors	2.5 (11)	1.4 (6)	0.9 (4)	0.0 (0)	0.0 (0)	0.0 (0)	0.0 (0)	0.2 (1)
Sell traditional beer and buy food with profit	2.3 (10)	0.7 (3)	1.4 (6)	0.2 (1)	0.0 (0)	0.0 (0)	0.0 (0)	0.0 (0)
Gather wild food, hunt, or harvest immature crops	2.1 (9)	1.4 (6)	0.5 (2)	0.0 (0)	0.0 (0)	0.0 (0)	0.0 (0)	0.2 (1)
Consume seed stock held for next season	2.1 (9)	0.9 (4)	0.5 (2)	0.5 (2)	0.0 (0)	0.0 (0)	0.0 (0)	0.2 (1)
Feed working members of HH at the expense of non-working members	2.1 (9)	1.2 (5)	0.5 (2)	0.2 (1)	0.2 (1)	0.0 (0)	0.0 (0)	0.0 (0)
Feed adult members of HH at the expense of children members	2.1 (9)	0.5 (2)	0.2 (1)	1.2 (5)	0.2 (1)	0.0 (0)	0.0 (0)	0.0 (0)
Feed male members of HH at the expense of female members	2.1 (9)	0.0 (0)	0.5 (2)	0.2 (1)	1.4 (6)	0.0 (0)	0.0 (0)	0.0 (0)

**Table 4:** Household food security characteristics over 30 days (*n* = 429).

Food Security Characteristics	%
*HFIAS domains*	
Anxiety and uncertainty over food	45.2
Insufficient food quality	40.2
Insufficient food intake	46.0
*HFIAS conditions*	
Worrying about food intake	43.7
Not able to eat preferred food	40.6
Limited variety of food	39.5
Eating unwanted foods	38.9
Eating smaller meals	33.1
Eating fewer meals	26.8
No food in the house	15.6
Sleeping hungry	8.2
The whole day and night without food	6.5
*HFIAS categories*	
Food secure	46.0
Mildly food insecure	23.8
Moderately food insecure	26.3
Severe food insecurity	4.0

**Table 5:** Determinants of household’s food insecurity (HFIAS).

Variables				Bivariate		Multivariate	
	Characteristics	N	HFI (%)	OR (CI)	*p* Value	AOR (CI)	*p* Value
Gender	Male	345	82.83	0.716 (0.444–1.155)	0.181	1.048 (0.561–1.957)	0.884
	Female	84	1.17				
Education	Illiterate	21	5.15	0.886 (0.365–2.150)	0.826	0.827 (0.259–2.642)	0.748
	Literate	408	94.85				
Farm ownership	Yes	29	7.30	0.829 (.386–1.780)	0.702	0.987 (0.410–2.379)	0.977
	No	400	92.70				
Field ownership	Yes	60	12.45	1.322 (0.765–2.282)	0.331	1.709 (0.866–3.374)	0.122
	No	369	87.55				
Home garden ownership	Yes	256	63.95	0.685 (0.465–1.011)	0.035	0.670 (0.405–1.110)	0.120
	No	172	36.05				
DDS	Poor dietary intake	240	53.65	1.227 (0.836–1.800)	0.329	1.370 (0.841–2.233)	0.206
	Good dietary intake	189	46.35				
Nutritional knowledge	Yes	363	81.97	1.576 (0.916–2.710)	0.064		
	No	66	18.03				
BMI	≤Normal	20	5.15	0.773 (0.309–1.931)	0.651	0.788 (0.269–2.309)	0.664
	>Normal	406	93.56				

**Table 6:** Biochemical indicators of households’ respondents (*n* = 429).

Variables	%	Mean	Std. Deviation	*p* Value
*Random blood glucose (mmol/L)*		9.7386	3.4863	<0.001
Normal (<11.1)	67.5			
Diabetes (>11.1)	32.5			
*Total blood cholesterol (mmol/L)*		3.6893	0.7421	<0.001
Normal (<5.0)	93.2			
At risk (<7.5)	6.8			

## Data Availability

Data supporting reported results can be accessed by communicating by email with the corresponding author.
